# X-ray Diffraction Analysis of ProRoot Mineral Trioxide Aggregate Hydrated at Different pH Values

**DOI:** 10.7508/iej.2016.02.007

**Published:** 2016-03-20

**Authors:** Hengameh Akhavan, Pooneh Mohebbi, Amir Firouzi, Mehdi Noroozi

**Affiliations:** a *Department of Endodontics, Dental Branch, Islamic Azad University, Tehran, Iran;*; b *Advanced Materials Center, Faculty of Materials and Metallurgy Engineering, Najafabad Branch, Islamic Azad University, Najafabad, Isfahan, Iran; *; c *Clinical Assistant Professor, University of British Columbia, Faculty of Dentistry, Vancouver BC, Canada*

**Keywords:** Mineral Trioxide Aggregate, pH, X-ray Diffraction

## Abstract

**Introduction:**

The aim of this study was to compare the chemical compounds of white ProRoot mineral trioxide aggregate (WMTA) hydrated at different pH environments.

**Methods and Materials:**

Mixed samples of WMTA were kept in acidic (pH=5.4), neutral (pH=7.4) and alkaline (pH=9.4) environments for 48 h. Then, X-ray diffraction (XRD) analysis was performed for both hydrated and powder forms of WMTA. Portlandite crystalline structures of environments were compared from three aspects: intensity (height of the peak, corresponding to the concentration), crystallinity (peak area/total area) and crystal size (full-width at half-maximum of the peak).

**Results:**

After matching the peaks of each sample with those of the International Center for Diffraction Data (ICDD) database, the main constituent of all set cements and powder form was found to be bismuth oxide. Acidic environment exhibited lower intensity and crystallinity of portlandite in comparison with neutral environment.

**Conclusion:**

The highest concentration and crystallinity of portlandite were observed in WMTA samples hydrated at neutral pH and the highest crystal size was detected after hydration in alkaline pH.

## Introduction

Mineral trioxide aggregate (MTA) is the gold standard endodontic biomaterial due to its biocompatibility, sealing ability and hard-tissue forming capacity [1]. Despite its numerous applications in dentistry and especially endodontics, one of the major drawbacks of MTA is the possible influence of different environmental pH values on the physical and chemical properties of the material [2]. 

Neutral environment is not present in all clinical situations. In cases of infection or calcium hydroxide therapy, acidic or alkaline environments are encountered, respectively [3, 4]. Some physical properties of ProRoot MTA, including the push out bond strength, surface hardness and setting time, have been evaluated in different pH environments [5-8]. Nevertheless, physical behavior of any biomaterial is a result of changes in its chemical aspect. Until now, the impact of acidic, neutral and alkaline pH values have not been comprehensively assessed on the chemical constituents of tooth-colored/white ProRoot MTA (WMTA).

When X-ray beams interact with a crystalline substance, some beams are diffracted with a specific intensity and angle, which is known as 2*θ*. Every crystalline particle always gives the same diffraction pattern, independent of the presence of other particles in the material. This is the fundamental of X-ray diffraction analysis (XRD). XRD studies have shown that the powder of MTA mainly consists of calcium silicate oxide and bismuth oxide [9] and the constituents of hydrated cement are mostly, calcium silicate oxide, bismuth oxide and portlandite (famously known as calcium hydroxide), with the latter being responsible for the bioactivity and biocompatibility of MTA [10]. However, it is possible that the final chemical constituents of set cement is different in variable environmental pH values during setting. 

The aim of this study was to compare the chemical compounds of WMTA hydrated at different pH environments.

**Figure 1:**
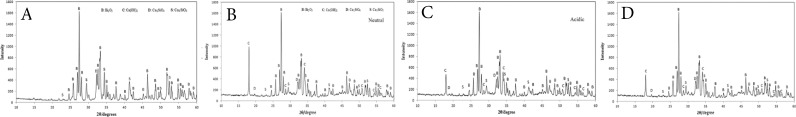
*A)* X-ray diffractogram of powder form of ProRoot MTA; *B) *X-ray diffractogram of hydrated MTA at neutral pH, *C) *X-ray diffractogram of hydrated MTA at acidic pH and* D) *X-ray diffractogram of hydrated MTA at alkaline pH

## Materials and Methods

White ProRoot MTA (Dentsply, Tulsa Dental, Tulsa, OK, USA; Batch number; 09003851) was mixed with water at a ratio of 3:1 by weight, as instructed by the manufacturer.

The mixed materials were packed into three resin cylindrical molds (8×10 mm) with a condenser. Three groups, each containing one specimen, were prepared in separate glass vials. Each glass vial was dedicated to specific pH environment. Neutral, acidic or alkaline pH were created by gauzes impregnated with distilled water (pH=7.4), distilled water buffered by butyric acid (pH=5.4) or distilled water buffered by potassium hydroxide (pH=9.4), respectively. The specimens were transferred to an incubator of 37̊C temperature for 48 h. To sustain the desired pH during the experimental time, the gauzes were kept in close contact to the cement surface and were replaced after 24 h. 

Both hydrated (mixed), and unhydrated (powder) cements were mounted for XRD analysis. The hydrated cements were milled into very fine powder. An automated x-ray diffractometer (X'Pert PRO MPD, Philips, Eindhoven, Netherlands) with Cu K*α* radiation was set at 40 kVp and 30 mA. Data were collected from scan range of 10-60 ˚2*θ* with a scan speed of 2^º^/min.

X-Manager evaluation software (Spectrum Viewer Basic 2.6.3) was used for the analysis. The peaks of each sample were matched with those of the International Center for Diffraction Data (ICDD) database (International Center for Diffraction Data, Newton square, PA, USA).

In order to find out the differences among cements hydrated at different environments, portlandite crystalline structures were compared in three aspects: intensity (height of the peak), crystallinity (peak area/total area) and crystal size [full-width at half-maximum (FMWH) of the peak].

## Results

**Figure 2:**
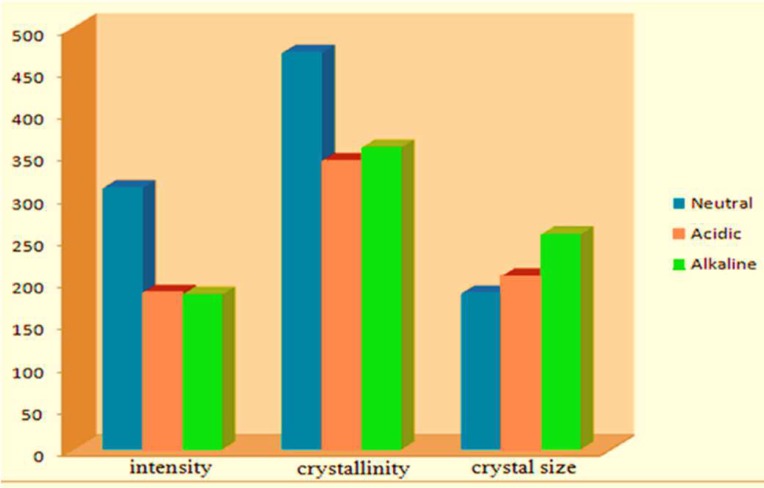
Comparison of intensity, crystallinity (multiplied by 10000) and crystal size (multiplied by 1000) of portlandite in ProRoot MTA at neutral, acidic and alkaline pH

The diffraction patterns of the set cements at different pH values and powder form are shown in Figures 1A to D, respectively. In powder form, the sharpest peak was related to bismuth oxide at 27.4 ˚2*θ*, followed by tricalcium silicate. In set cements, bismuth oxide had the sharpest peak. Peaks related to tri- and dicalcium silicate reduced in intensity in the hydrated phases. Portlandite was identified in the set forms at 18 ˚ and 34 ˚ 2*θ*. However, it was not observed in the powder forms. The outcome of comparison of portlandite crystalline structures is shown in Figure 2.

## Discussion

XRD analysis is a method to identify the compound composition of the material. Crystalline phases can be detected even in unknown samples. The x-ray diffraction pattern of a pure substance is, therefore, a fingerprint of the substance [9].

The intensity of each phase in XRD pattern is proportional to the phase concentration [10]. The major disadvantage of XRD analysis for Portland cement and MTA is the superimposition of the peaks and the presence of multiple compounds inside the materials. An example of this superimposition is observed at 52̊ 2θ for bismuth oxide, dicalcium silicate and tricalcium silicate compounds [10].

MTA hydration produces calcium silicate hydrates (CSH) and portlandite [11]. Although, CSH was not identifiable in hydrated MTA due to small crystal size [12] or amorphous structure [10], phases representing portlandite were detected. After hydration, the peaks of tri- and dicalcium silicate reduced in intensity in all three acidic, alkaline and neutral environments, which indicates dissolving of C-S reactants in water and formation of hydration byproducts.

Production of portlandite causes MTA to be highly reactive and makes it susceptible to interact with dental materials, body fluids and blood. Hydroxyapatite is formed due to the reaction of portlandite with phosphate present within the body fluids [13, 14]. Bioactivity and biocompatibility of MTA are induced by releasing portlandite [10, 15]. 

In the present study, concentration and crystallinity of portlandite were highest in neutral environment in comparison with acidic and alkaline conditions. This finding has been inferred from previous studies. Cements hydrated at acidic pH, have less surface microhardness [8], less compressive strength [16] and less push-out bond strength [7] and also more porosity, eroded cubic-shaped crystals and absence of needle-like crystals (ettringite) under scanning electron microscope (SEM) [12]. Studies evaluating cements hydrated at alkaline pH, showed less push out bond strength at pH levels of 9.4 and 10.4 in comparison with neutral pH [6] and less surface microhardness, more porosity and unhydrated structure in MTA exposed to pH level of 10.4 [5]. In this study, larger crystal size of portlandite in acidic condition in comparison with neutral environment may be due to the fusion of the adjacent crystals together.

In WMTA also small amounts of tricalcium aluminate was reported which resulted in low production of ettringite and monosulphate [10]. Tricalcium aluminate has the fastest hydration among MTA components. This accelerates the hydration process and improves the compressive strength of tricalcium silicate/tricalcium aluminate compounds comparing pure tricalcium silicate [14]. 

In our study, bismuth oxide levels did not differ in hydrated and unhydrated samples of white MTA, which was in contrast with the results reported by Camilleri [10], who found active participation of bismuth oxide in hydration mechanism of MTA .

For future studies, it is recommended to use solutions more similar to clinical conditions such as simulated body fluid (SBF) or phosphate buffered solution (PBS). In addition, for obtaining more reliable quantitative measurements, using internal controls such as rutile is recommended.

## Conclusion

The highest value of intensity and crystallinity of portlandite crystal/compound was seen after white ProRoot MTA was hydrated at neutral pH and the largest crystal size was formed in an alkaline environment.
